# Successful Extra-anatomical Management of a Salmonella-Associated Mycotic Pseudoaneurysm of the Left Common Iliac Artery

**DOI:** 10.7759/cureus.84255

**Published:** 2025-05-16

**Authors:** Almukhtar Almomatten, Zainab A Alammar, Mohammed I Almomatten, Ali Alsalman

**Affiliations:** 1 Department of Vascular Surgery, King Fahad Hospital Al Hofuf, Hofuf, SAU; 2 Department of General Surgery, King Fahad Hospital Al Hofuf, Hofuf, SAU

**Keywords:** antibiotic therapy, aortic aneurysm, extra-anatomical bypass, iliac artery pseudoaneurysm, mycotic pseudoaneurysm, pseudoaneurysm, salmonella infection, synthetic graft, vascular infection management

## Abstract

Mycotic pseudoaneurysms, rare but life-threatening vascular conditions, result from infections of arterial walls, often involving Salmonella species. This case report details a 62-year-old man with diabetes presenting with a Salmonella-induced mycotic pseudoaneurysm of the left common iliac artery (CIA). The patient exhibited severe abdominal and radiating back pain, initially suggestive of gastrointestinal or urinary pathology. Diagnostic imaging revealed a thrombosed pseudoaneurysm with associated inflammatory changes, including wall enhancement and fat stranding, necessitating urgent intervention. Surgical management included extra-anatomical femorofemoral bypass with a polytetrafluoroethylene graft, ligation of the infected artery, and angioplasty of the right CIA to restore blood flow. Postoperative care with tailored antibiotic therapy, based on culture results, ensured the successful resolution of the infection and the prevention of complications. The patient achieved full recovery, with follow-up imaging confirming graft patency and stability of the aortic aneurysm. This case underscores the importance of early diagnosis, a multidisciplinary approach, and combined surgical and antibiotic strategies in managing mycotic pseudoaneurysms. It highlights the need for further research into optimizing management protocols for these complex infections.

## Introduction

A mycotic pseudoaneurysm is a type of infected false aneurysm usually caused by bacterial infection, leading to arterial wall disruption and balloon-like dilation [1]. They develop due to a preexisting infectious arteritis rather than direct trauma. Like other infectious processes, they may occur at injection sites or due to hematogenous seeding. Mycotic aneurysms are false aneurysms and are prone to rupture due to instability. They may also progress to form arteriovenous fistulae and produce septic emboli [2]. Despite being a life-threatening condition with high risks of rupture, sepsis, and mortality, early detection of infected pseudoaneurysms remains challenging. The management strategy for mycotic-infected pseudoaneurysms is controversial due to the complexity and severity of the condition [2-4].

## Case presentation

A 62-year-old Saudi male patient, known to have diabetes mellitus, hypertension, and benign prostatic hypertrophy, with a history of a previous hospital admission for chronic pancreatitis, presented to the emergency department with a severe epigastric and left iliac fossa (LIF) pain radiating to the lower back that increases with bowel motion, and found to be associated with dysuria for 10 days.

The patient appeared ill, tachycardic, and dehydrated (Table 1). Abdominal examination revealed severe LIF and epigastric tenderness, with no distention, visible mass, or signs of generalized peritonitis. Bilateral femoral pulses were weak.

**Table 1:** Patient's vital signs upon emergency department presentation SpO_2_: peripheral capillary oxygen saturation

Vitals	Normal values	Patient's values
Heart rate	60-100 beat/minute	110 beat/minute
Blood pressure	120/80 mmHg	141/71 mmHg
Temperature	36.5-37.4°C	37°C
Respiratory rate	12-20 per/minute	18 per/minute
SpO_2 _(on room air)	94% or more	95%

**Figure 1:**
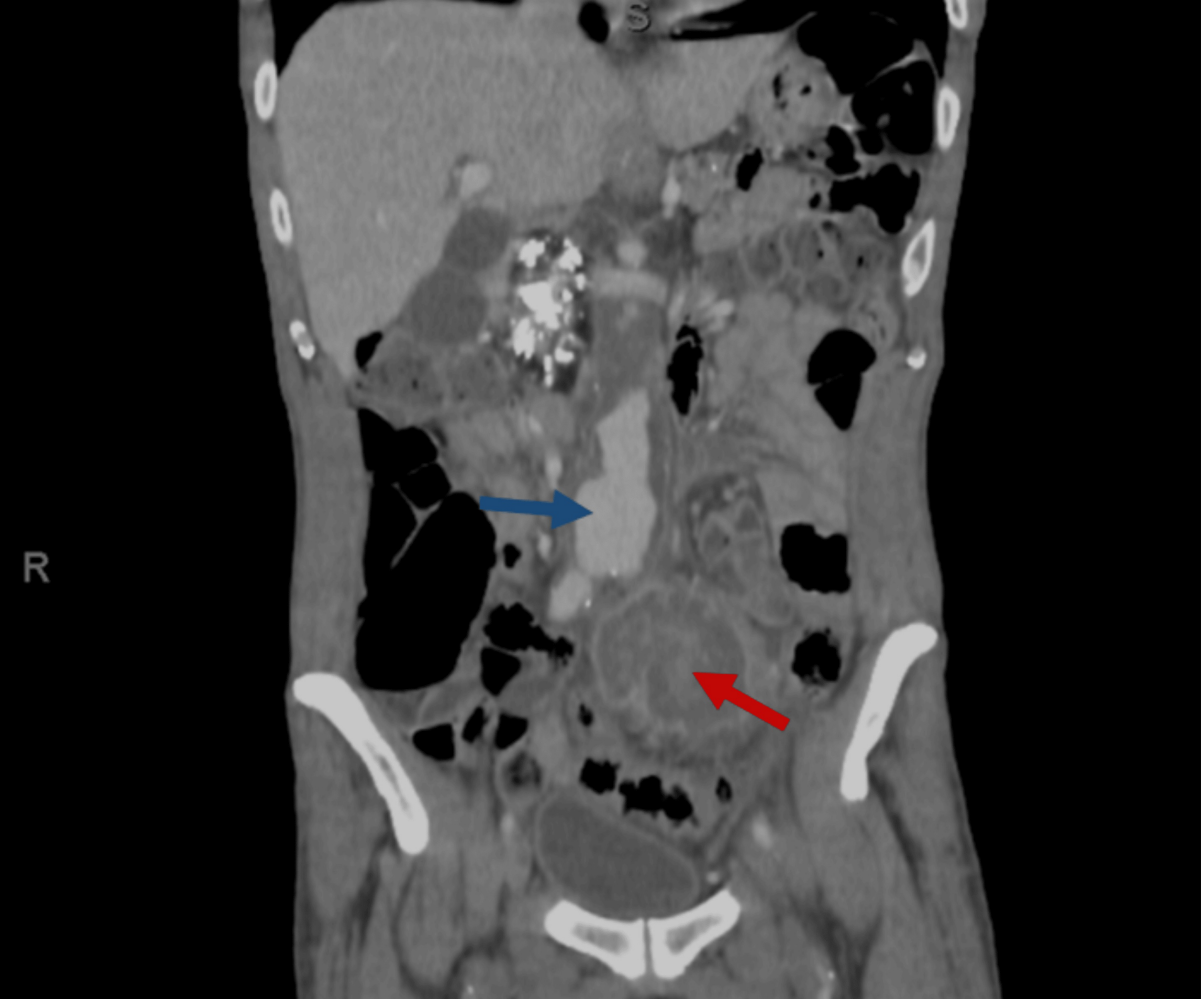
Coronal view of the computed tomography image. The red arrow indicates the left iliac artery pseudoaneurysm, and the blue arrow indicates abdominal aorta aneurysm

On the day of the presentation, the labs showed mild leukocytosis with a white blood count of 10.64 × 10⁹/L (normal range, 4-10 × 10⁹/L) and a hemoglobin level of 6.5 g/dL (normal range, 13-17 g/dL). The cause of anemia was not formally investigated, but it was presumed to be anemia of chronic disease. Abdominal computed tomography (CT) with intravenous (IV) contrast showed a moderate amount of intraperitoneal fluid, multilaminated thrombosed pseudoaneurysm arising from the left common iliac artery (CIA) measuring 4.9 x 6.6 x 6.4 cm (Figure 1), with a partially occlusive thrombosis causing luminal narrowing. In addition, there was a fusiform aneurysm of the abdominal aorta just above the bifurcation with a diseased atheromatous wall. Features of chronic pancreatitis include multiple calcifications and a large stone noted within the pancreatic head. There is mild left-sided hydronephrosis with delayed excretion of the contrast; the left ureter is closely related to the left CIA pseudoaneurysm, which could be causing compression. There are positive findings of a moderate intra-abdominal collection.

The patient was admitted to the intensive care unit for vital stabilization, and blood cultures were taken. Crossmatch revealed a blood group of O positive. The patient received one unit of packed red blood cells, was kept nothing per oral (PO), and a glucose-insulin-potassium protocol was initiated. IV broad-spectrum antibiotics (meropenem 1 g every eight hours) were initiated. Anesthesia, general internal medicine, and urology consultations were done for preoperative evaluation and preparation.

Operative note

The patient was positioned supine under general anesthesia. Prepping and draping were performed using standard sterile techniques, and left ureteric catheterization was completed.

A lower midline laparotomy incision was made, and free bloody fluid was suctioned and sent for culture. The small bowel was retracted to the right and the large bowel cephalad to expose the left CIA. A large, contained rupture of the left CIA with significant blood clots was identified.

**Figure 2:**
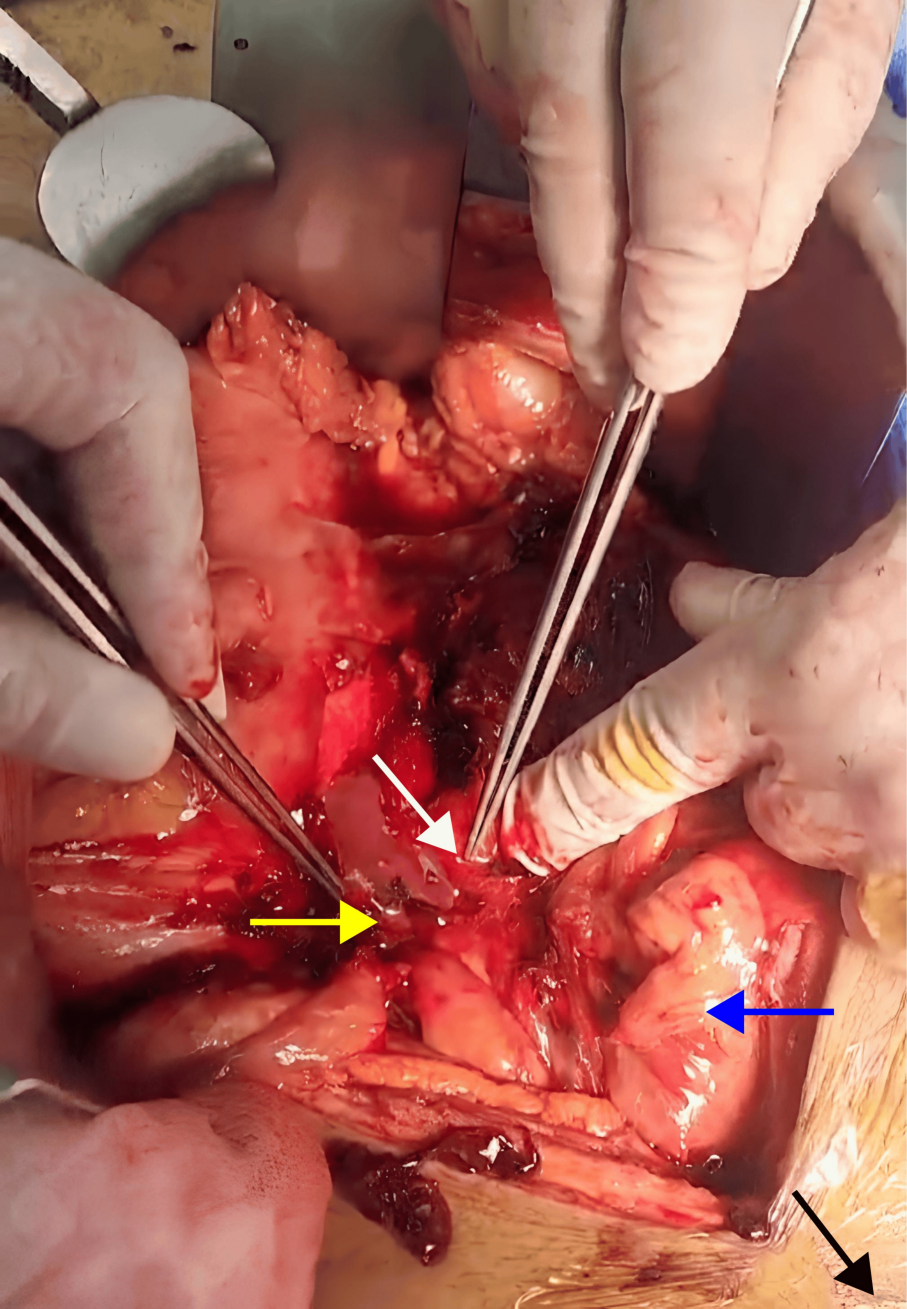
Intraoperative exposure of the infected left common iliac artery. The black arrow indicates the direction of the head (cephalad), the white arrow indicates the left CIA with surrounding pus, the yellow arrow indicates the right CIA, and the blue arrow indicates the small bowel CIA: common iliac artery

Before manipulating the pseudoaneurysm, proximal aortic and distal CIA control was established to allow clamp application if necessary. The pseudoaneurysm was carefully dissected, and a large number of clots were evacuated. This led to the release of pus from a multiseptated sac (Figure 2), which was suctioned, washed, and sent for tissue and swab cultures.

Due to the presence of frank infection and disintegrated tissue, the decision was made to ligate the proximal and distal ends of the ruptured left CIA and perform an extra-anatomical femorofemoral (fem-fem) bypass. After aortic clamping, CIA ligation was performed, followed by clamp release.

The right femoral artery pulse was absent after ligation, prompting an aortic endarterectomy proximal to the bifurcation. Ruptured atheromatous material was removed, followed by right CIA balloon angioplasty (8 mm × 40 mm). Restoration of the right femoral pulse was confirmed.

A synthetic 7-mm polytetrafluoroethylene (PTFE) graft was used for the fem-fem bypass, achieving good blood flow with triphasic signals and intact bilateral lower limb perfusion. Drains were placed in the left abdominal cavity, pelvis, and left femoral region. The wound was closed, and the patient was shifted to the recovery area.

Postoperative course

On postoperative day 1, the patient showed significant improvement, with stable vital signs, well-controlled pain, and no signs of systemic infection. Palpable pulses were present in both lower limbs, and Doppler ultrasonography confirmed biphasic signals in the bilateral femoral, popliteal, anterior and posterior tibial, and dorsalis pedis arteries. A strong thrill was palpable over the graft site.

On postoperative day 2, the patient was transferred to the general ward. Cultures identified Salmonella group D infection (nontyphoidal), and antibiotics were adjusted to ceftriaxone 2 g IV once daily and Bactrim^TM^ double strength (DS; sulfamethoxazole 800 mg and trimethoprim 160 mg; AR Scientific, Inc., Philadelphia, PA) PO twice daily for six weeks. An echocardiogram ruled out infective endocarditis, revealing normal cardiac function.

The patient was discharged safely five days postoperatively. At follow-up in the outpatient clinic over two months, the patient reported no active complaints and was found to be in good health. A follow-up CT angiography performed two months postoperatively showed no peritoneal fluid, resolution of the pseudoaneurysm, and no evidence of new aneurysms. The fem-fem bypass graft was functioning well.

## Discussion

Mycotic pseudoaneurysms are rare, life-threatening vascular conditions that arise due to infection of the arterial wall, leading to the formation of a pseudoaneurysm. The term "mycotic" originally referred to fungal infections but has since broadened to include bacterial etiologies, with *Staphylococcus aureus* and Salmonella species being the most common culprits [3,5]. Although the incidence of Salmonella-associated mycotic pseudoaneurysms is uncommon, they are particularly significant due to the aggressive nature of the infection and a high mortality rate, often exceeding 50% if not promptly diagnosed and treated [3,6].

Pathogenesis and risk factors

Salmonella, particularly nontyphoidal strains, can invade atherosclerotic plaques, leading to endothelial disruption and arterial wall infection [2,3,7]. This process is facilitated by predisposing conditions such as diabetes mellitus, chronic renal failure, and immunosuppressive therapy, which compromise the host's immune response and vascular integrity [2,3,8]. In the present case, the patient’s history of diabetes mellitus, which is known to impair both innate and adaptive immune responses, likely contributed to increased susceptibility to Salmonella infection and the subsequent development of a mycotic pseudoaneurysm. Pathogenesis involves bacterial seeding in the arterial wall, typically in the context of bacteremia, as evidenced by the positive blood cultures of Salmonella group D [5,9].

Clinical presentation and diagnostic challenges

The clinical presentation of mycotic pseudoaneurysms is often insidious, with symptoms that may mimic more common conditions such as urinary tract infections or gastrointestinal disorders, leading to delays in diagnosis [3,5,10]. In this case, the patient presented with severe epigastric and left iliac fossa pain, which, along with radiating back pain and dysuria, could easily have been mistaken for other abdominal pathologies. The subtlety of initial symptoms underscores the need for a high index of suspicion, particularly in patients with known risk factors and atypical pain patterns [11-13].

Diagnostic imaging plays a pivotal role in identifying mycotic pseudoaneurysms. In this case, a contrast-enhanced CT scan revealed a thrombosed pseudoaneurysm of the left CIA, with features suggestive of an ongoing infection, including peripheral wall enhancement [14]. This imaging modality remains the gold standard for diagnosing mycotic pseudoaneurysms, providing detailed information on the extent of the aneurysm, the involvement of surrounding structures, and any associated complications such as thrombosis or rupture [5,11,15,16].

Management strategies: surgical and antibiotic therapy

The management of mycotic pseudoaneurysms is complex and necessitates a multidisciplinary approach. Surgical intervention is generally required to prevent rupture and manage infection [15,17]. Infected pseudoaneurysms are typically managed through in situ or extra-anatomic bypass grafting, often accompanied by surgical debridement to control the infection [3,8,11]. In this case, the extra-anatomical fem-fem bypass, combined with ligation of the infected artery, was chosen to effectively manage the infection while ensuring proper distal perfusion. This approach is particularly advantageous in cases where direct reconstruction might pose a high risk of reinfection or is technically unfeasible due to extensive infection [18].

In addition to surgical intervention, long-term antibiotic therapy is essential to eradicate the underlying infection [8,19]. The antibiotics are typically tailored based on culture results, with ceftriaxone and Bactrim^TM^ DS being appropriate choices in this case, given the sensitivity profile of the isolated Salmonella strain [7,20]. The patient received a six-week course of targeted antibiotic therapy. Broad-spectrum antibiotics, such as meropenem, were initiated empirically to cover a wide range of potential pathogens until specific culture results were available [21].

Justification for fem-fem bypass

In this particular case, the choice of a fem-fem bypass was driven by the extent of the infection and the condition of the left CIA. Upon accessing the left CIA, we encountered a significant infection surrounding the artery, with a large sac containing blood clots mixed with pus. This indicated severe infection and vascular compromise, making direct arterial reconstruction highly risky. Placing a graft in such a contaminated field could have led to reinfection and subsequent graft failure [1]. Therefore, the decision was made to ligate the proximal and distal ends of the ruptured left CIA to control the infection and prevent further contamination.

An extra-anatomical fem-fem bypass was performed using a synthetic graft (non-rifampin-soaked PTFE). This approach allowed for the revascularization of the lower limbs without having to place a graft directly in the infected area. This method also mitigated the risk of reinfection while ensuring that adequate blood flow was restored to both lower extremities. In addition, an aortic endarterectomy and right CIA angioplasty were performed to address the absent pulse in the right femoral artery, further stabilizing the patient’s vascular status. The final outcome was confirmed by good blood flow with triphasic signals through the graft, and the patient’s lower limb perfusion remained intact throughout the procedure.

The patient’s postoperative course, characterized by significant improvement and successful revascularization without signs of recurrent infection, further supports the efficacy of this surgical strategy.

Comparative outcomes and prognostic considerations

The outcomes of mycotic pseudoaneurysms are heavily influenced by the timeliness of diagnosis, the choice of surgical technique, and the adequacy of antibiotic therapy. While the mortality rate remains high, early intervention significantly improves prognosis [14,22]. In this case, the patient’s favorable outcome, characterized by a successful surgical procedure and uneventful recovery, highlights the effectiveness of combining aggressive surgical management with targeted antibiotic therapy [23].

Comparatively, cases involving delayed diagnosis or suboptimal surgical management have been associated with poor outcomes, including recurrent infection, graft failure, and even fatal complications such as sepsis or hemorrhage [21]. For instance, a similar case of a Salmonella-induced mycotic pseudoaneurysm managed with endovascular stenting demonstrated the potential for graft infection and late-stage complications, emphasizing the importance of vigilant postoperative monitoring [24].

## Conclusions

Managing mycotic pseudoaneurysms, particularly those caused by Salmonella, remains a formidable challenge due to the aggressive nature of the infection and the critical need for timely intervention. This case underscores the importance of maintaining a high index of suspicion in at-risk patients, the pivotal role of imaging in early diagnosis, and the necessity of a coordinated surgical and medical approach. While open surgical repair remains the standard in extensively infected cases, endovascular treatment with prolonged antibiotic (six-week course) therapy is an emerging alternative in select patients. This highlights the need for greater clinical vigilance in immunocompromised individuals and those with vascular pathology. Future research should focus on refining both open and endovascular strategies, along with optimizing antimicrobial regimens, to improve outcomes in this high-risk population.

The successful treatment of this case not only adds to the growing body of literature on mycotic pseudoaneurysms but also provides valuable insights into the management of complex vascular infections, paving the way for better therapeutic protocols and improved patient survival.
